# Supplementary material for: Parasympathetic and Sympathetic Activity Are Associated with Individual Differences in Neural Indices of Selective Attention in Adults
Source: Psychophysiology. Author manuscript; Available in PMC 2025 Nov 20. (PMC12634002; doi:10.1111/psyp.13079)
Supplement: 1 [file NIHMS2116066-supplement-1.pdf]

bioRxiv preprint doi: https://doi.org/10.1101/173377; this version posted August 8, 2017. The copyright holder for this preprint (which was not certified by peer review) is the author/funder, who has granted bioRxiv a license to display the preprint in perpetuity. It is made available under aCC-BY 4.0 International license.

Supplemental Results

Due to the small number of males (n=6) in the overall sample, results reported in the main manuscript are replicated here with females only (N=87). Any differences in findings between the two analyses are summarized in a final section, at the end of the results below.

Characterizing physiological reactivity to the selective attention task

Paired-sample *t*-tests of baseline and task values for HF-HRV, PEP, heart rate, and respiration rate showed significant task reactivity for all measures. HF-HRV power declined from baseline (M = 6.12, SD = 1.12) to task (M = 6.00, SD = 1.08), $t(86)$ = 2.44, p = .017. PEP values shortened from baseline (M = 113.57, SD = 10.08) to task (M = 112.40, SD = 10.28), $t(86)$ = 2.45, p = .016. These changes in HF-HRV and PEP were associated with concurrent slowing of heart rate from baseline (M = 73.61, SD = 10.39) to task (M = 72.27, SD = 9.80), $t(86)$ = 3.22, p = .002, and acceleration of respiration rate from baseline (M = 15.82, SD = 1.84) to task (M = 17.33, SD = 2.35), $t(86)$ = -7.54, p < .001.

Characterizing effects of selective attention on ERPs

For P1 mean amplitudes, results revealed an interaction of attention x laterality, $F(2, 172)$ = 3.95, p = .023, such that significant effects of attention were seen at right-lateralized electrode clusters (p = .033) but not at left-lateralized or midline clusters (*p*s > .57). Follow-up comparisons showed that significant effects of attention on P1 amplitudes at the group level were seen at the right medial (p = .019) and right posterior (p = .047) electrode clusters.

For N1 mean amplitudes, results revealed a main effect of attention, $F(1, 86)$ = 8.06, p = .006, as well as an interaction of attention x laterality, $F(2, 172)$ = 12.61, p < .001, such that significant effects of attention were seen at left-lateralized (p = .002) and midline electrode clusters (p < .001) but not at the right-lateralized clusters (p = .588). Follow-up comparisons demonstrated significant attention effects on N1 amplitudes broadly across the scalp [left anterior, p = .015; central anterior, p = .012; left central, p = .024; central midline, p = .001; left posterior, p = .001; midline posterior, p < .001].

Associations between ANS and ERP effects of selective attention

Correlations amongst all variables of interest for subsequent analyses are shown in Supplemental Table 1. Below, these results and follow-up analyses are presented separately for each ERP component. Notably, there were no significant associations between socioeconoimc risk factors and any of the autonomic or ERP measures. Therefore, socioeconomic risk was not included in the following analyses.

P1 component (100-150 ms). No significant associations were observed between effects of attention on P1 amplitudes and measures of HF-HRV [baseline HF-HRV: $r(84)$ = .08, p = .478; task HF-HRV: $r(84)$ = .02, p = .885; HF-HRV reactivity: $r(84)$ = -.14, p = .199] or measures of PEP [baseline PEP: $r(84)$ = .07, p = .525; task PEP: $r(84)$ = .03, p = .818; PEP reactivity: $r(84)$ = -.10, p = .364].

bioRxiv preprint doi: https://doi.org/10.1101/173377; this version posted August 8, 2017. The copyright holder for this preprint (which was not certified by peer review) is the author/funder, who has granted bioRxiv a license to display the preprint in perpetuity. It is made available under aCC-BY 4.0 International license.

N1 component (175-225 ms). A significant association was observed between effects of selective attention on N1 amplitudes and baseline PEP, $r(84) = .23$, $p = ..035$, with a similar effect observed between N1 amplitudes and task values of PEP, $r(84) = .27$, $p = .014$. There was a marginal trend of N1 amplitudes correlating with baseline HF-HRV, $r(84) = -.21$, $p = .058$. Shorter baseline PEP and higher baseline HF-HRV were associated with a larger effect of selective attention on N1 amplitudes. Notably, the directionality of these effects remained when controlling for respiration rate [baseline PEP, $r(83) = .22$, $p = .044$; baseline HF-HRV, $r(83) = -.20$, $p = .072$][3]. Follow-up analyses of attended and unattended ERPs separately suggested that the relationship between baseline physiology and the N1 attention effect was driven by associations specific to attended ERPs. Shorter baseline PEP was associated with larger negative amplitudes to attended ERPs, $r(83) = .23$, $p = .034$, and although higher baseline HF-HRV was associated with larger negative amplitudes to attended ERPs, this relationship was non-significant, $r(83) = -.18$, $p = .106$. There was no evidence of associations between baseline physiology and unattended ERPs (*p*s > .85).

Linear regression models were then run to clarify the join contributions of baseline HF-HRV and baseline PEP with effects of selective attention on N1 amplitudes, while controlling for reactivity values and age. As shown in Supplemental Table 2, the effect of attention on N1 amplitudes was significantly predicted by baseline HF-HRV and baseline PEP [$R^2 = .14$, $F(3, 83) = 3.62$, $p = .016$], with unique variance accounted for by baseline HF-HRV [$\beta = -.23$, $p = .045$] and baseline PEP [$\beta = .23$, $p = .027$]. Adding reactivity values of HF-HRV and PEP did not contribute additional explained variance to the model [R^2 change = .02, F change(2, 81) = .96, $p = .387$]. To test for interactions between baseline HF-HRV and PEP, an additional model was performed including an interaction term of baseline HF-HRV x baseline PEP, but adding the interaction term did not contribute additional explained variance [R^2 change = .03, F change(1, 80) = 2.50, $p = .118$].

Summary of analyses after excluding male participants

Overall, there was a high degree of overlap between results for the full sample (N=93) and results for the sample when only females were considered (N=87). The same pattern of physiological reactivity was observed across HF-HRV, PEP, heart rate, and respiration rate, with very similar raw values for each measure between the two samples. Analyses of the attention effect at the P1 and N1 components were nearly identical, with the lone exception being an additional electrode cluster showing a significant N1 attention effect with the female-only sample (left central, $p = .024$), which was only marginally associated in the full sample (left central, $p = .058$).

Associations between the ERP attention effect, HF-HRV, and PEP were also very similar to analyses with the full sample. Both analyses showed no relationship between

[3] Effects of selective attention on N1 amplitudes were not significantly associated with baseline heart rate ($p = .437$), heart rate reactivity ($p = .553$), baseline respiration rate ($p = .814$), or respiration rate reactivity ($p = .397$).

bioRxiv preprint doi: https://doi.org/10.1101/173377; this version posted August 8, 2017. The copyright holder for this preprint (which was not certified by peer review) is the author/funder, who has granted bioRxiv a license to display the preprint in perpetuity. It is made available under aCC-BY 4.0 International license.

HF-HRV or PEP with the P1 attention effect, while baseline HF-HRV and PEP showed associations with the N1 attention effect. Although the association between baseline HF-HRV and the N1 attention effect was relatively attenuated in the female-only sample, $r(84) = -.21$, $p = .058$, relative to the full sample, $r(90) = -.22$, $p = .037$, when entering both baseline HF-HRV and baseline PEP into the model together with age, baseline HF-HRV remained a significant predictor of the N1 attention effect along with baseline PEP.

Supplemental Table 1. *Partial Correlations Controlling for Age for Female Participants (N=87).*

	1	2	3	4	5	6	7	8	9	10	11	12
1. Socioeconomic risk	-											
2. HF-HRV baseline	.06	-										
3. HF-HRV task	-.03	.88***	-									
4. HF-HRV reactivity	-.17	-.34**	.15	-								
5. PEP baseline	-.03	.06	.11	.09	-							
6. PEP task	-.09	.04	.07	.07	.90***	-						
7. PEP reactivity	-.13	-.05	-.08	-.06	-.30**	.16	-					
8. Attended P1 amp.	.06	-.05	-.11	-.10	.17	.07	-.22*	-				
9. Unattended P1 amp.	.17	-.15	-.15	.03	.08	.04	-.09	.32**	-			
10. Att. – Unatt. P1 amp.	-.09	.08	.02	-.12	.08	.03	-.12	.62***	-.54***	-		
11. Attended N1 amp.	.04	-.19t	-.16	.08	.25*	.29**	.07	.03	<.01	.03	-	
12. Unattended N1 amp.	.10	.03	.02	-.02	.01	-.02	-.02	-.16	.20t	-.30**	.46***	-
13. Att.- Unatt. N1 amp.	-.06	-.22*	-.18	.09	.24*	.31**	.08	.17	-.18	.30**	.57***	-.46***

***, *p* < .001; **, *p* < .01; *, *p* < .05; t, *p* < .07

Supplemental Table 2. *Regression Models Predicting the Effect of Selective Attention on N1 Amplitudes from Baseline HF-HRV and PEP for Female Participants (N=87).*

Model 1			Model 2			Model 3		
	β	*p*		*β*	*p*		*β*	*p*
Age	.04	.736	Age	.02	.844	Age	-.01	.962
**Baseline PEP**	**.23**	**.027***	**Baseline PEP**	**.26**	**.019***	**Baseline PEP**	**.23**	**.033***
**Baseline HF-HRV**	**-.23**	**.045***	Baseline HF-HRV	-.22	.059	**Baseline HF-HRV**	**-.24**	**.043***
			PEP reactivity	.15	.170	PEP reactivity	.15	.177
			HF-HRV reactivity	.20	.842	HF-HRV reactivity	.07	.569
						Baseline PEP x HF-HRV	-.17	.118
Model fit, $F(3, 83) = 3.62$, $p = .016$*			Model fit, $F(5, 81) = 2.56$, $p = .034$*			Model fit, $F(6, 80) = 2.59$, $p = .024$*		
$R^2 = .116$			F change $(2, 81) = .96$, $p = .387$			F change $(1, 80) = 2.50$, $p = .118$		
			$R^2 = .136$			$R^2 = .162$		
